# Coexpression of PD-L1/PD-1 with CXCR3/CD36 and IL-19 Increase in Extranodal Lymphoma

**DOI:** 10.1155/2023/4556586

**Published:** 2023-01-23

**Authors:** Manal Mohamed Saber

**Affiliations:** Department of Clinical Pathology, Faculty of Medicine, Minia University, Minia 61519, Egypt

## Abstract

Many studies have demonstrated that PD-L1/PD-1 signaling is an immune evasion mechanism in tumors. PD-L1/PD-1 coexpression with CXCR3/CD36 in peripheral lymphocytes in lymphoma still needs to be clarified. The current study investigated PD-L1/PD-1 coexpression with CXCR3/CD36 in circulating lymphocytes, serum IL-19 levels, and their correlation with clinical outcome and extranodal involvement in lymphoma. *Subjects and Methods*. The coexpression of PD-L1/PD-1 with CXCR3/CD36 on circulating lymphocytes was analyzed by flow cytometry in 78 lymphoma patients before and after therapy and in 50 healthy controls. The concentration levels of IL-19 in serum were assessed by an ELISA. *Results*. PD-L1 and PD-1 were expressed on circulating CXCR3+ and CD36+ lymphocytes in lymphoma and were significantly higher in patients with extranodal involvement than in lymphoma patients without extranodal involvement (*P* < 0.001). Elevated IL-19 levels were observed in lymphoma patients and increased significantly in extranodal involvement (*P* < 0.001). High percentages of PD-L1+CXCR3+ and PD-1+CXCR3+ lymphocytes were associated with high LDH levels, hepatomegaly, lymphedema, advanced tumor stage, and recurrence. Furthermore, patients with splenomegaly and generalized lymphadenopathy had high percentages of PD-L1+CXCR3+ lymphocytes. In addition, levels of PD-L1/PD-1 coexpression with CXCR3 and IL-19 were significantly associated with bone marrow, lung, and lymph vessel involvement. Further analysis revealed that high percentages of PD-L1+CD36+ and PD-1+CD36+ lymphocytes were associated with lung and bone marrow involvement. Patients with high levels of PD-L1/PD-1 coexpression with CXCR3 and IL-19 had inferior event-free survival (EFS) compared with that in lymphoma patients with low levels. EFS was decreased in patients with high percentages of PD-L1+CD36+ and PD-1+CD36+ lymphocytes. When using the receiver operating characteristic (ROC) curve, the superiority of IL-19 (area under the curve (AUC): 0.993) and PD-L1+CXCR3+% (AUC: 0.961) to PD-1+CXCR3+% (AUC: 0.805), PD-L1+CD36+% (AUC: 0.694), and PD-1+CD36+% (AUC 0.769) was evident in the diagnosis of extranodal involvement, identifying lymphoma patients with extranodal involvement from patients without extranodal involvement. *Conclusions*. Coexpression of PD-L1/PD-1 with CXCR3/CD36 in circulating lymphocytes and serum IL-19 levels contributes to poor prognosis and might be potential markers for extranodal involvement in lymphoma.

## 1. Introduction

Lymphomas are heterogeneous hematological malignancies arising in the lymphatic and reticuloendothelial systems. Hodgkin lymphoma (HL) and non-Hodgkin lymphoma (NHL) are the two forms of lymphoma [1, 2]. Both HL and NHL can impact any organ or tissue in the body. Extranodal lymphoma describes the lymphomatous invasion of tissues other than lymphoid organs or lymph nodes. The most commonly affected organs by lymphomatous infiltration are the lung, liver, bone, stomach, skin, spleen, central nervous system, and Waldeyer's ring [3–6]. Depending on the lymphoma stage and the histological type, the distribution and prevalence of the affected organs var**y** [7, 8]. The incidence of extranodal involvement is increasing. The most significant challenges in lymphoma therapy are recurrence and extranodal involvement [9].

T cells express the regulatory inhibitory protein programmed death-1 (PD-1), a transmembrane element [10]. Macrophages, T lymphocytes, B lymphocytes, regulatory cells, and tumor cells express PD-ligand 1 (PD-L1) [11]. PD-L1/PD-1 transmits restraining signals to T cells in lymphomas, causing functional exhaustion, anergy, or lymphocyte apoptosis [12, 13]. Previous studies reported overexpression of PD-L1/PD-1 in HL and various forms of NHL [14, 15]. However, few studies have investigated the relationship between PD-L1/PD-1 and extranodal involvement in lymphoma. PD-L1/PD-1 antibodies have launched a new era of lymphoma immunotherapy [16].

CXCR3, a G protein-coupled cell surface receptor (GPCR), is present on CD4+ and CD8+ lymphocytes surface and other cells, like epithelial cells [17]. Still, interestingly, FOXp3+ regulatory T cells express it (Tregs) [18–20]. CXCR3 expression is upregulated in lymphoma [21, 22]. CXCR3 expression initiates tumor cell survival and metastasis through CXCR3 ligand expression [23–25].

Cluster differentiation 36 (CD36) is a scavenger receptor on the surface of monocytes, adipocytes, dendritic cells, macrophages, and lymphocytes [26–33]. CD36 can bind to many ligands, including fatty acids, thrombospondin 1, and apoptotic cells [34]. CD36 functions include molecular adhesion, apoptosis, immune identification, and lipid uptake [35–37]. CD36 expression is associated with tumor cell growth and metastases [38, 39]. In an orthotopic OSCC mouse model, Pascual et al. showed that the suppression of CD36 prevented lymph node metastasis, demonstrating the necessity of fatty acids and CD36 for lymph node metastasis [34].

Interleukin-19 (IL-19) is a member of the IL-10 family [40]. Essential sources of IL-19 are macrophages, monocytes, B lymphocytes, epithelial, and endothelial cells [41]. High serum IL-19 levels have been detected in patients with NHL [42]. High IL-19 levels are associated with poor prognosis, metastasis, and advanced tumor stages [43, 44].

Tumors have developed various strategies, disrupting “immune checkpoints” to get through the host's immune system [45]. The study assessed PD-L1/PD-1 coexpression with CXCR3/CD36 in circulating CXCR3 and CD36 positive lymphocytes and serum IL-19 levels in extranodal lymphoma. Furthermore, the study investigated the effect of this coexpression and IL-19 on lymphoma prognosis. This study could help uncover new mechanistic insights into the extranodal involvement of lymphoma and assess a new era of lymphoma immunotherapy.

## 2. Subjects and Methods

### 2.1. Subjects

A total of 78 lymphoma patients and 50 healthy volunteers participated in the study. Healthy controls included thirty-six males and fourteen females. Healthy volunteers' range in age was from 26 to 70 years old. Seventy-eight lymphoma subjects ranged in age from 11 to 81 years, including 39 men and 39 women. Patients received treatment as soon as the primary diagnosis is confirmed. Patients with lymphoma were followed, and the patients were separated into two groups based on how well they responded to treatment: group I with no extranodal involvement and group II with extranodal involvement. Lymphoma patients' clinical outcomes were evaluated according to Cheson response criteria (National Cancer Institute Working Group standards for response to treatment) [46]. According to established criteria, the effectiveness of the treatment was assessed in 78 patients, of whom twenty-nine had a complete response (CR), eighteen had a partial response (PR), twenty-seven had a recurrence, and four had a treatment failure [46]. Pretherapy and posttherapy blood samples were taken to assess IL-19 levels and PD-L1/PD-1 coexpression with CXCR3/CD36 in peripheral lymphocytes for the presence of extranodal involvement and response to treatment. Patients with missing pathological or clinical information on their lymphoma were excluded from the study. Atherosclerosis, various cancers, and persistent infections were all ruled out. Each subject signed an informed consent form. This study was performed in the Clinical Pathology Department, Faculty of Medicine, Minia University.

### 2.2. Clinical Data

A complete clinical examination was performed on all subjects to monitor lymphadenopathy and hepatosplenomegaly, and detailed history questionnaires were completed. To determine the performance, type, and stage of lymphoma, all patients underwent bone marrow aspiration and lymph node biopsy. The Ann Arbor classification has been considered for clinical staging [47]. The modified International Workshop on CLL (iwCLL) 2018 criteria were used in diagnosis [48], and CLL stages were assessed according to the Rai staging system [49]. Three expert pathologists validated all pathological specimens following WHO criteria [50]. Using a flow cytometer, the patients' immunophenotyping was performed. All patients obtained chest X-rays and pelviabdominal ultrasounds to rule out extramedullary involvement. The patient's performance was evaluated according to Eastern Cooperative Oncology Group scale [51]. PET (positron emission tomography) or CT (computed tomography) scans were used. The Ann Arbor approach [52] was used to assess lymphoma staging. BCR-ABL, and hepatitis C virus (HCV) RNA, was assessed by quantitative RT-PCR.

### 2.3. Follow-Up and Lymphoma Therapy

Follow-up for lymphoma patients was performed at the hematology clinic and by telephone. The posttherapy lymphoma status was evaluated in lymphoma patients by means of clinical examination and PET/CT scans. Patients will be evaluated every three months for the identification of lymphoma progression. The follow-up was only for 78 patients with lymphoma out of 92 patients. The follow-up could not be performed for these patients as some patients were referred to other centers, some have died, and some had rejected blood samples rewithdrawal. Event-free survival (EFS) measures the time between the end of therapy and the commencement of an event (extranodal involvement). Seven follicular cell lymphoma (FCL) and twenty-eight diffuse large B cell lymphoma (DLBCL) patients' subjects had CHOP therapy (cyclophosphamide, hydroxydaunorubicin, oncovin, and prednisone) [53]. In contrast, those with HL had ABVD therapy (Adriamycin, bleomycin, vinblastine, and dacarbazine) and radiation [54]. Seven patients with chronic lymphocytic leukemia (CLL) underwent various treatments designed based on the therapy indications, stage, and comorbidities. The following strategies were used for CLL: no therapy (approach of watching and waiting if there is no therapy indication); corticosteroids (quiescent CLL with immune cytopenias); FCR chemotherapy (fludarabine, cyclophosphamide, and rituximab) was indicated for CLL progressive illness [49]. One marginal zone lymphoma (MZL) patient and two mucosa-associated lymphoid tissue (MALT) lymphoma patients had chemotherapy, rituximab, and radiotherapy.

### 2.4. Blood Sampling

All subjects provided peripheral samples, which were taken under very sterile conditions. Eight milliliters of blood were collected. For a complete blood count (CBC), 2 mL of blood was put into an EDTA tube. For flow cytometric analysis, 2 mL of blood was inserted into an EDTA tube. A plain tube was filled with 4 mL of blood, then centrifuged for 5 minutes at 3000 rpm. Serum was isolated and used to analyze liver function tests (bilirubin, albumin, alanine aminotransferase (ALT), and aspartate aminotransferase (AST)), lactate dehydrogenase (LDH), kidney function tests (serum creatinine and blood urea), and random blood sugar. The leftover serum was kept at -70°C until the serum IL-19 concentrations were measured.

### 2.5. Laboratory Investigations

A computerized hematology analyzer was used to determine CBC (Celtac G, Nihon Kohden Corporation, Japan). Renal function analysis (blood urea nitrogen and serum creatinine), liver function analysis (ALT, AST, bilirubin, and albumin), and random blood sugar were performed using an autoanalyzer (SELECTRA, ELITech Group, clinical chemistry automation systems, France). An automated ACE chemistry analyzer assessed LDH (Schiapparelli Biosystems. INC; USA).

### 2.6. Immunophenotyping

PD-1/PD-L1 coexpression with CXCR3/CD36 was determined in collected blood samples. To identify the various immune cells, the following human monoclonal antibodies were used, as directed by the manufacturer: PD-1 (BioLegend, catalog No. 329906), CXCR3 monoclonal antibody (BioLegend; catalog no. 353704), PD-L1 (BioLegend, catalog No. 309706), and CD36 monoclonal antibody (BioLegend, catalog No. 336204). Unstained cells were utilized as a negative control for every patient. Negative isotypic controls were performed using other tubes. As isotype controls, monoclonal PE-conjugated IgG2a (BioLegend; catalog no. 402203) and FITC IgG1 were used (BioLegend; catalog no. 400110).

### 2.7. Staining Flow Cytometric Analysis

The percentages of PD-L1+CXCR3+, PD-1+CXCR3, PD-L1+CD36+, and PD-1+CD36+ lymphocytes were calculated using flow cytometry BD FACSCanto II (Becton Dickinson, CA, San Diego, USA). In brief, a hundred microliters of anticoagulated-EDTA whole blood were stained with five uL of monoclonal antibodies and incubated at room temperature in the dark for twenty minutes. The cells were lysed using the lysing buffer and set aside for about ten minutes at room temperature in the dark. Cells were then washed two times with PBS and resuspended in 300 uL of PBS solution. A minimum of 10,000 events were analyzed. Lymphocyte gating was carried out through the FSC/SSC plots (front scatter vs. side scatter technique) [55–60], and then, CXCR3+ and CD36+ lymphocytes were gated for PDL1/PD-1 expression [61–65]. The cutoff values were calculated using the isotypic controls as a guide. Unstained cells were employed as a negative control for every patient.

### 2.8. ELISA for IL-19 Analysis

The concentrations of IL-19 in serum were assessed using an ELISA kit (Sunred Biological Technology Co., Ltd., Shanghai) following the manufacturer's instructions. In the 96 wells of the microtiter strips, a specific monoclonal for IL-19 was coated (sensitivity for IL-19: 1.3 pg/mL). A microtiter plate reader was used to determine optical densities at 450 nm.

### 2.9. Statistical Analyses

The data were analyzed by applying the SPSS application (Statistical Package for Social Sciences) version 25. Normally, quantitative data was analyzed by minimum and maximum range and mean and standard deviation (SD). The median and interquartile range (IQR) was utilized for quantitative nonparametric data, while percentage and number were employed for categorical data. Mann–Whitney analysis was carried out to analyze quantitative nonparametric data between two groups. Kruskal-Wallis test was carried out to analyze nonparametric data between more than two groups, proceeded by pairwise comparisons between each two groups applying Bonferroni correction. Fisher's exact test, or the Chi-square test, was carried out to compare the qualitative data between groups. Association between continuous and qualitative ordinal variables was assessed by Spearman's correlation, while Pearson's correlation was performed for the association between 2 continuous variables.

The Kaplan-Meier analysis was carried out to assess EFS, comparing the survival curves using the log-rank test. The variables' cutoff point, area under the curve (AUC), specificity, and sensitivity were calculated using the receiver operator characteristic (ROC) curve. *P* values less than 0.05 were considered significant.

## 3. Results

### 3.1. Subjects

The study included 50 healthy individuals and 78 lymphoma patients. Normal controls included thirty-six males and fourteen females. Healthy volunteers ranged in age from 24 to 81 years old. Seventy-eight lymphoma subjects ranged in age from 11 to 81 years, including 39 males and 39 females. The criteria for all subjects are listed in (Supplementary Table 1).

After therapy and follow-up, there were 34 lymphoma patients with extranodal involvement and 44 without extranodal involvement. Patients with extranodal involvement had a mean age of 42.81 ± 7.6, ranging from 11 to 81, while those without extranodal involvement had a mean age of 42 ± 16.1, ranging from 18 to 75. Supplementary Table 2 reveals no significant differences regarding age, lymphoma types, and subtypes between patients without extranodal involvement (*N* = 34) and patients with extranodal involvement (*N* = 34). Lymphoma patients with and without extranodal involvement revealed statistical significance regarding sex (*P* = 0.039), stage (*P* < 0.001), recurrence (*P* < 0.001), and death (*P* = 0.018). Among lymphoma subjects with extranodal involvement, 73.5% had a recurrence, and 20.6% died. 26.5% of patients with extranodal involvement presented with stage III diseases, while 58.8% had stage IV diseases. Only 2.9% of patients with extranodal involvement had stage I, and 11.8% had stage II (Supplementary Table 2).

The different extranodal involvement sites in lymphoma patients are shown in Table 1. Bone marrow involvement and lymph vessel infiltration were found in 33.3% and 21.8% of patients, respectively. 12.8% of patients presented with lung involvement, while approximately 10.3% had spleen infiltration. Furthermore, 9% of lymphoma patients showed liver involvement. Only 1.3% of patients had involvement of the intestine, thyroid, or central nervous system (Table 1).

### 3.2. High PD-L1/PD-1 Expression in Circulating CD36+ and CXCR3+ Lymphocytes in Newly Diagnosed Lymphoma Patients

Flow cytometric analysis investigated CD36 and CXCR3 expression in peripheral lymphocytes in 78 newly diagnosed lymphoma patients and 50 healthy controls. Lymphocyte gating was shown in Supplementary Figures 1-4. CD36 and CXCR3 positive lymphocytes were found in small percentages in healthy controls (median: 4.2% (range: 3.5–5.7) and median: 11.2% (range: 9.7–13.2)). High pretherapy CXCR3+% and CD36+% of cells were observed in lymphoma patients than in normal volunteers (median: 40% vs. 11.2%; 13% vs. 4.2%, *P* < 0.001) (Table 2).

The study then examined whether peripheral CD36 and CXCR3 positive lymphocytes expressed PD-L1 and PD-1. Compared to healthy controls, newly diagnosed lymphoma patients had higher PD-L1+CXCR3+% and PD-1+CXCR3+% (32% vs. 1.1%; 10% vs. 0.7%, *P* < 0.001). Interestingly, PD-L1+CD36+% and PD-1+CD36+% in newly diagnosed lymphoma subjects were significantly higher than in healthy volunteers (median: 8% vs. 1%; 5 vs. 0.2, *P* < 0.001) (Table 2).

### 3.3. Coexpression of PD-L1/PD-1 with CXCR3+/CD36 Defines Extranodal Involvement in Lymphoma

**Figure 1:**
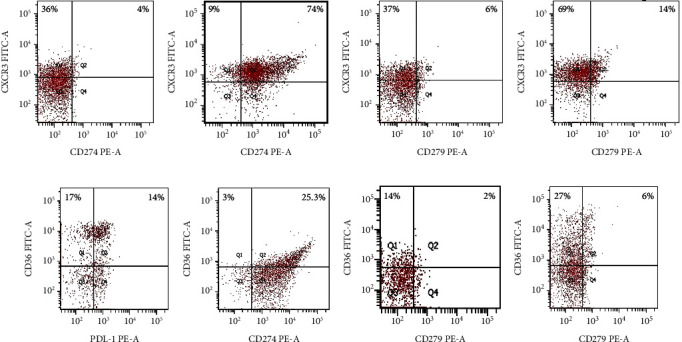
PD-L1/PD-1 is coexpressed with CXCR3/CD36 on peripheral lymphocytes in extranodal involvement. (a, b) Dot plots of PD-L1+CXCR3+% from a lymphoma patient without and with extranodal involvement. (c, d) Dot plots of PD-1+CXCR3+% from a lymphoma patient without and with extranodal involvement. (e, f) Dot plots of PD-L1+CD36+% from a lymphoma patient without and with extranodal involvement. (g, h) Dot plots of PD-1+CD36+% from a lymphoma patient without and with extranodal involvement. The percentages of positive cells are shown in the upper right quadrant.

Extranodal involvement of lymphoma indicates an inferior prognosis for lymphoma. PD-L1/PD-1 coexpression with CXCR3/CD36 in circulating lymphocytes was investigated in posttherapy lymphoma patients with extranodal involvement (*n* = 34) or without extranodal involvement (*n* = 44) (Figure 1). Posttherapy 34 lymphoma patients with extranodal involvement were characterized by higher percentages of peripheral CXCR3 positive lymphocytes than those in posttherapy 44 patients without extranodal involvement (median: 78% vs. 26%, *P* < 0.001) (Figure 1). When considering circulating CD36+ lymphocytes, CD36+% was lower in extranodal involvement compared with subjects without extranodal involvement (median: 27% vs. 55.5%, *P* < 0.001) (Figure 1).

Lymphoma subjects with extranodal involvement had higher PD-L1+CXCR3+% and PD-1+CXCR3+% compared with subjects without extranodal involvement (median 65% vs. 4%; 12.2% vs. 2%, *P* < 0.001, respectively) (Figures 1(a)–1(d)). Furthermore, PD-L1+CD36% and PD-1+CD36+% were significantly higher in subjects with extranodal involvement compared with patients without extranodal involvement (median 22 vs. 8, *P* = 0.004; 5.8% vs. 1%, *P* < 0.001) (Figures 1(e)–1(h)).

A significant increase in posttherapy PD-L1/PD-1 coexpression with CXCR3/CD36 in circulating lymphocytes was detected in the extranodal involvement group compared with the pretherapy samples of the same group (Supplementary Table 3). However, lymphoma patients without extranodal involvement had a lower posttherapy PDL-1/PD-1 coexpression with CXCR3/CD36 than in the pretherapy samples of the same patients (Supplementary Table 4).

### 3.4. IL-19 in Lymphoma Patients

**Figure 2:**
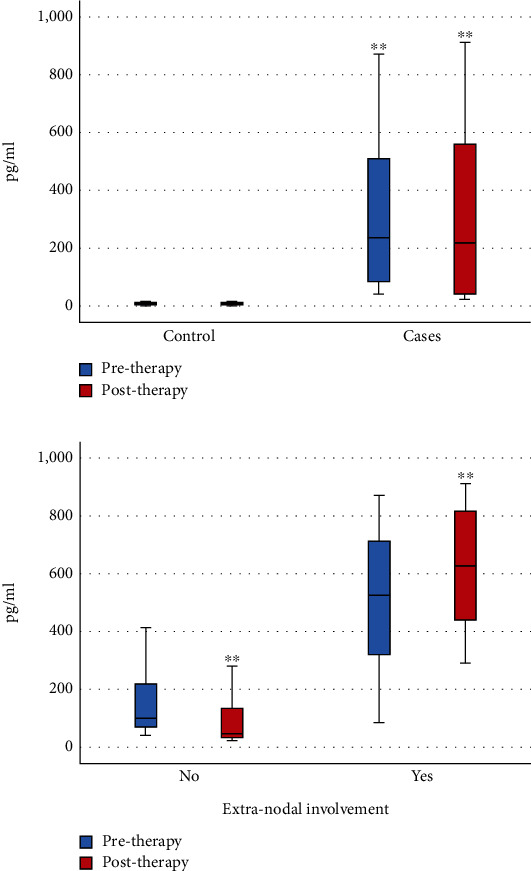
IL-19 concentrations in healthy volunteers and lymphoma patients. (a) Pretherapy and posttherapy IL-19 levels in 78 lymphoma patients and 50 healthy volunteers. (b) Pretherapy and posttherapy IL-19 levels in patients without and with extranodal involvement. ^∗∗^ identifies high significant differences at *P* < 0.001.

Newly diagnosed 78 lymphoma patients had significantly higher IL-19 levels than normal volunteers (median = 237 vs. 7.2 pg/mL, *P* < 0.001). Moreover, lymphoma patients had higher posttherapy IL-19 levels than the normal controls (median: 219 vs. 7, *P* < 0.001) (Figure 2(a)).

Posttherapy IL-19 levels were detected in lymphoma patients with extranodal involvement, and the median level was 628 pg/mL (range: 439-817.3), which was significantly higher than that of subjects without extranodal invasion with a median of 46.5 pg/mL (range: 33-137.5) (*P* < 0.001) (Figure 2(b)). Interestingly, compared to patients without extranodal involvement, lymphoma patients with extranodal involvement had higher pretherapy IL-19 levels (median: 525 vs. 100, *P* < 0.001) (Figure 2(b)).

### 3.5. Coexpression of PD-L1/PD-1 with CXCR3+/CD36 and IL-19 Defines Lymphoma Clinical Outcome

Patients with CR had significantly lower pretherapy IL-19, CXCR3+%, and PD-L1+CXCR3+% compared to the PR, recurrence, and refractory groups (*P* < 0.05). Furthermore, compared to the PR, recurrence, and refractory groups, the CR group had a significant reduction in posttherapy IL-19, CXCR3+%, PD-L1+CXCR3+%, and PD-1+CXCR3+% (*P* < 0.05). Additionally, PD-L1+CD36+% and PD-1+CD36+% were significantly lower in subjects with CR compared to the other groups. Contrarily, comparing the CR group to the other groups, there was a substantial rise in posttherapy CD36+% (*P* < 0.001, *P* < 0.001, and *P* = 0.010) (Table 3).

The CR group showed a significant reduction in pretherapy PD-1+CXCR3+% and posttherapy PD-L1+CXCR3+% compared to the PR group and recurrence group (*P* < 0.001). However, no significant difference was observed when subjects with CR were compared to the treatment-refractory patients (*P* = 0.092 and *P* = 0.055). In addition, the CR patients' PD-L1+CD36+% and PD-1+CD36+ % were significantly lower than those in the PR group (*P* = 0.037 and *P* = 0.002). However, no significant difference was detected between the CR group and the recurrence group or the refractory group (*P* > 0.05) (Table 3).

Pretherapy IL-19 levels, CXCR3+%, and PD-L1+CXCR3+% in lymphoma patients with recurrence were statistically higher than in the PR patients (*P* < 0.001). Additionally, the recurrence group's posttherapy IL-19 levels, PD-L1+CXCR3+, and PD-1+CXCR3+% were significantly higher than those of PR patients (*P* = 0.018, *P* = 0.013, and *P* = 0.011) (Table 3). Furthermore, pretherapy PD-1+CD36+% and posttherapy CD36+% in patients with recurrence were significantly lower than in PR patients (*P* = 0.022 and *P* = 0.002). However, no significant differences were observed when the treatment-refractory group compared to PR or recurrence groups (*P* > 0.05) (Table 3).

### 3.6. Correlation between PDL-1/PD-1 Coexpression with CXCR3/CD36, IL-19, and Laboratory and Clinical Criteria

The results revealed a positive association between pretherapy CXCR3+%, PD-L1/PD-1+CXCR3%, and LDH (*r* = 0.344, *P* = 0.002; *r* = 0.375, *P* = 0.001; *r* = 0.315, *P* = 0.005, respectively). However, negative associations between CXCR3+%, PD-L1/PD-1+CXCR3%, and albumin levels were identified (*r* = −0.269; *P* = 0.017; *r* = −0.326, *P* = 0.004; *r* = −0.337, *P* = 0.003, respectively). Additionally, there was a negative association between PD-L1+CXCR3+% and hemoglobin (*r* = −0.254, *P* = 0.025) (Table 4).

CXCR3+%, PD-L1+CXCR3+%, and PD-1+CXCR3+% were positively correlated with hepatomegaly (*r* = 0.464, *P* < 0.001; *r* = 0.398, *P* < 0.001, *r* = 0.335, *P* = 0.003). Data revealed that CXCR3+%, PD-L1+CXCR3+%, and PD-1+CXCR3+% positively correlated with lymphoma stages (*r* = 0.498, *P* < 0.001; *r* = 0.437, *P* < 0.001, *r* = 0.333, *P* = 0.002) (Table 4). Moreover, CXCR3+% and PD-L1+CXCR3+% associated positively with both splenomegaly (*r* = 0.348, *P* = 0.002; *r* = 0.268, *P* = 0.018) and general lymphadenopathy (*r* = 0.362, *P* = 0.001; *r* = 0.304, *P* = 0.007). Furthermore, CXCR3+% and PD-L1+CXCR3+% positively correlated with BCR-ABL (*r* = 0.262, *P* = 0.020; *r* = 0.271, *P* = 0.016). Additionally, CXCR3+%, PD-L1+CXCR3+%, and PD-1+CXCR3+% had a significant association with lymphoma recurrence (*r* = 0.645, *P* < 0.001; *r* = 0.676, *P* < 0.001; *r* = 0.310, *P* = 0.006) (Table 4). CD36+%, PD-L1+CXCR3+%, and PD-1+CD36+% were associated with some of clinical parameters but without significance (*P* > 0.05) (Table 4).

Interestingly, pretherapy IL-19 was positively correlated with LDH levels (*r* = 0.349 and *P* = 0.002), hepatomegaly (*r* = 0.362 and *P* = 0.001), and splenomegaly (*r* = 0.231 and *P* = 0.042). Furthermore, pretherapy IL-19 levels were significantly associated with lymphedema, general lymphadenopathy, and recurrence (*r* = 0.426, *P* < 0.001; *r* = 0.239, *P* = 0.035; *r* = 0.641, *P* < 0.001) (Table 4).

### 3.7. Coexpression of PD-L1/PD-1 and CXCR3/CD36 and IL-19 Is Associated with Extranodal Involvement

Posttherapy CXCR3+%, PD-L1+CXCR3+%, and PD-1+CXCR3+% were positively associated with extranodal involvement (*r* = 0.771, *P* < 0.001; *r* = 0.793, *P* < 0.001; *r* = 0.528, *P* < 0.001). CXCR3+%, PD-L1+CXCR3+%, and PD-1+CXCR3+% had a positive association with bone marrow involvement (*r* = 0.645, *P* < 0.001; *r* = 0.676, *P* < 0.001; *r* = 0.369, *P* < 0.001). Moreover, CXCR3+%, PD-L1+CXCR3+%, and PD-1+CXCR3+% were positively associated with lymph vessel involvement (*r* = 0.499, *P* < 0.001; *r* = 0.487, *P* < 0.001; *r* = 0.272, *P* = 0.016). Additionally, a significant association between CXCR3+%, PD-L1+CXCR3+%, PD-1+CXCR3+%, and lung involvement was identified (*r* = 0.277, *P* = 0.014; *r* = 0.286, *P* = 0.011; *r* = 0.235, *P* = 0.038) (Table 5).

Posttherapy PD-L1+CD36+% and PD-L1+CD36+% were positively correlated with extranodal involvement (*r* = 0.333, *P* = 0.003; *r* = 0.469, *P* < 0.001), while CD36+% had a negative association with extranodal involvement (*r* = −0.792 and *P* < 0.001). Of interest, PD-L1+CD36+% and PD-1+CD36+% were positively correlated with bone marrow infiltration (*r* = 0.312, *P* = 0.005; *r* = 0.390, *P* < 0.001). PD-1+CD36+% was positively correlated with lung involvement (*r* = 0.234; *P* = 0.040). CD36% was negatively correlated with bone marrow and lymph vessel involvement (*r* = −0.633, *P* < 0.001; *r* = −0.386, *P* < 0.001). Furthermore, percentages of CD36+ lymphocytes were correlated with spleen infiltration (*r* = 0.327; *P* = 0.004) (Table 5).

IL-19 concentrations and extranodal involvement were positively correlated (*r* = 0.848; *P* < 0.001). IL-19 levels are positively associated with bone marrow and lymph vessel involvement (*r* = 0.637, *P* < 0.001; *r* = 0.437, *P* < 0.001). Similarly, a positive association between posttherapy IL-19 levels and lung involvement was identified (*r* = 0.278; *P* = 0.014) (Table 5).

### 3.8. Prognostic Value of PD-L1/PD-1 Coexpression with CXCR3/CD36 and IL-19

**Figure 3:**
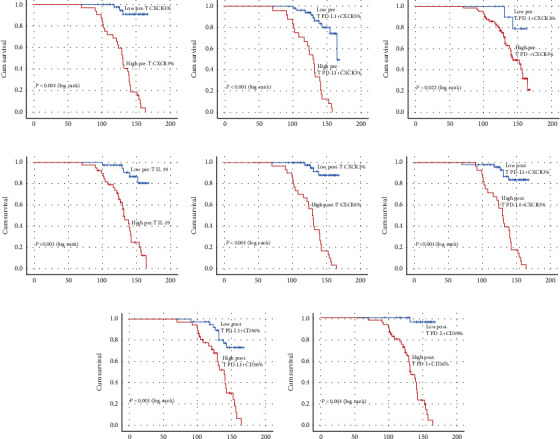
Kaplan-Meier analysis of EFS for IL-19 and PD-L1/PD-1 coexpression with CXCR3/CD36 status. (a) Pretherapy CXCR3+ lymphocytes. (b) Pretherapy PD-L1+CXCR3+ lymphocytes. (c) Pretherapy PD-1+CXCR3+ lymphocytes. (d) Pretherapy IL-19. (e) Posttherapy CXCR3+ lymphocytes. (f) Posttherapy PD-L1+CXCR3+ lymphocytes. (g) Posttherapy PD-1+CXCR3+ lymphocytes. (h) Posttherapy PD-1+CD36+ lymphocytes.

The effect of the markers on EFS was assessed using Kaplan-Meier statistics (Figure 3). Survival tree analysis identified that CXCR3+ and PD-L1+CXCR3+ staining percentages more than 50% were considered high, whereas those ≤50% were regarded as low staining. PD-1+CXCR3+ and PD-L1+CD36+ staining percentages more than 7% were considered high, whereas those ≤7% were regarded as low staining.

There was a high reduction in EFS in lymphoma patients with high percentages of pre- and posttherapy CXCR3+ lymphocytes (*P* < 0.001) (Figures 3(a) and 3(e)). Moreover, pre- and posttherapy PD-L1+CXCR3+ lymphocytes were negatively associated with EFS (*P* < 0.001) (Figures 3(b) and 3(f)). Patients' EFS was significantly decreased when their initial PD-1+CXCR3+ lymphocyte percentages were high (*P* = 0.022) (Figure 3(c)). High pre-T IL-19 levels were associated with inferior EFS (*P* < 0.001) (Figure 3(d)). A prolonged EFS was predicted by low posttherapy PD-L1+CD36+% and PD-1+CD36+% (*P* < 0.001) (Figures 3(g) and 3(h)).

The effect of the immune markers on overall survival and recurrence-free survival was determined using Kaplan-Meier statistics (data not shown). Patients with low pre- and posttherapy CXCR3+%, PD-L1+CXC3+%, and PD-1+CXCR3+% do better than those with a high percentage. Furthermore, low PD-L1+CD36+% and PD-L1+CD36+% predicted a more prolonged survival and recurrence-free time.

### 3.9. Diagnostic Utility of PD-L1/PD-1 Coexpression with CXCR3/CD36 and IL-19 in Identifying Extranodal Involvement

**Figure 4:**
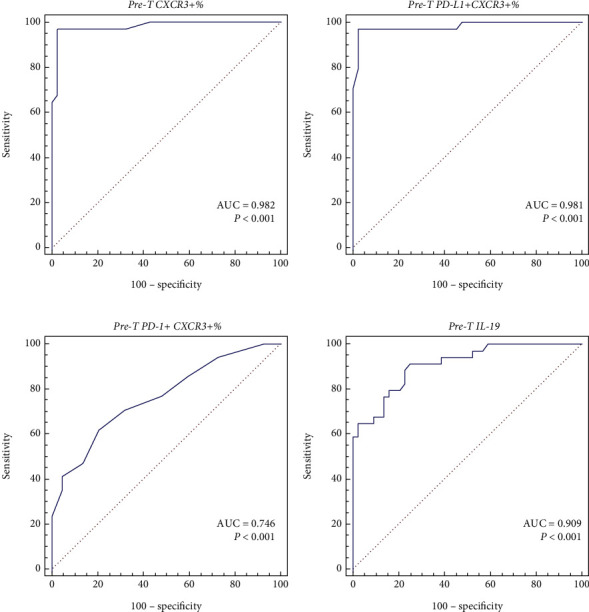
ROC curves of PD-L1/PD-1 coexpression with CXCR3/CD36 and IL-19 to predict extranodal involvement in lymphoma. (a) Pretherapy CXCR3+%. (b) Pretherapy PD-L1+CXCR3+%. (c) Pretherapy PD-1+CXCR3%. (d) Pretherapy IL-19.

Figure 4 shows the ROC analysis for predicting extranodal involvement in lymphoma. Pretherapy CXCR3+%, PD-L1+CXCR3+%, and PD-1+CXCR3+% could predict extranodal involvement with AUCs of 0.982, 0.981, and 0.764 (95% CI = 0.922 − 0.999; 0.921–0.999; 0.654-0.853; *P* < 0.001, respectively) (Figures 4(a)–4(c)). Pretherapy CXCR3+%, PD-L1+CXCR3+%, and PD-1+CXCR3+% cut-offs were >41, >33, and >11, respectively. Moreover, pretherapy CXCR3+%, PDL-1+CXCR3+%, and PD-1+CXC3+% sensitivities were 97.06%, 97.06%, and 61.67%, and the specificities were 97.73%, 97.73%, and 79.55%. Pretherapy CXCR3+% and PDL-1+CXCR3+ had the best sensitivity and specificity. Pretherapy IL-19 levels could predict extranodal involvement with an AUC of 0.909 (95% CI = 0.822 − 0.962, *P* < 0.001). The pretherapy IL-19 cut-off was >209 pg/mL, with specificity and sensitivity 75% and 91.18% (Figure 4(d)).

**Figure 5:**
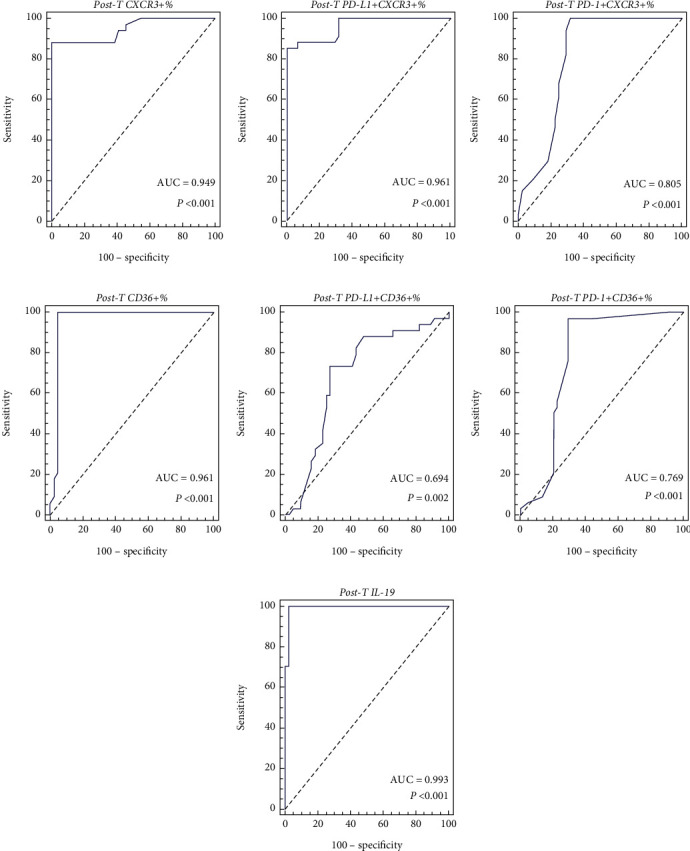
ROC curves of PD-L1/PD-1 coexpression with CXCR3/CD36 and IL-19 to diagnose extranodal involvement in lymphoma. (a) Posttherapy CXCR3+%. (b) Posttherapy PD-L1+CXCR3+%. (c) Posttherapy PD-1+CXCR3%. (d) Posttherapy CD36+%. (e) Posttherapy PD-L1+CD36%. (f) Posttherapy PD-1+CD36%. (g) Posttherapy IL-19.

Posttherapy CXCR3+%, PDL-1+CXCR3+%, and PD-1+CXC3+% could diagnose lymphoma patients with extranodal involvement. Posttherapy CXCR3+%, PDL-1+CXCR3+%, and PD-1+CXC3+% AUCs were 0.949, 0.961, and 0.805 (*P* < 0.001) at a cutoff >49, >40, and >3, respectively (Figures 5(a)–5(c)). Posttherapy PD-1+CXCR3+% had the best sensitivity of 100%, while PD-L1+CXCR3+% and CXCR3+% had the best specificity of 100%.

Moreover, the AUC values of the posttherapy CD36+%, PDL-1+CD36+%, and PD-1+CD36+% for extranodal involvement diagnosis were 0.961, 0.694, and 0.769 (95% CI = 0.890 − 0.992; 0.579-0.793; 0.659-0.857, respectively). Posttherapy CD36+%, PDL-1+CD36+%, and PD-1+CD36+% had cutoff levels ≤37, > 11, and>3 (*P* < 0.001, *P* = 0.002, and *P* < 0.001). Posttherapy CD36+% had the best sensitivity of 100% and the best specificity of 95.45% (Figures 5(d)–5(f)).

The posttherapy IL-19 AUC was 0.993 (95% CI = 0.941–1.000, *P* < 0.001), with a cutoff >280 pg/mL for diagnosis of extranodal involvement. IL-19 showed 100% sensitivity and 97.73% specificity (Figure 5(g)).

## 4. Discussion

Previous research found various immune evasion pathways in lymphomas, persuading that deception from antitumor immunity was required for the pathogenesis of lymphoma [66]. PD-L1/PD-1 signaling is one way cancers bypass the immune system [67]. PD-L1/PD-1 signaling has a major role in lymphocyte malfunction. However, anti-PDL1/PD-1 antibodies do not consistently reverse this mechanism, suggesting that other molecules may contribute to lymphocyte depletion [68]. The study assessed PD-L1/PD-1 expression in circulating CXCR3 and CD36-positive lymphocytes in lymphoma.

In this study, the percentage of peripheral CXCR3 and CD36 positive lymphocytes differed significantly between lymphoma patients and healthy controls. In earlier studies, investigations revealed that CXCR3 expression was restricted to activated T lymphocytes [69] and that CXCR3 and CD36 expression was detected in small percentages of peripheral lymphocytes [33, 70]. Other studies found that individuals with various forms of lymphoma had a higher percentage of peripheral CD36 and CXCR3+ lymphocytes, implying that lymphoma patients have an immunological defect [71, 72].

PD-L1/PD-1 coexpression with CXCR3/CD36 in peripheral lymphocytes has not been studied in lymphoma. In lymphomas, coexpression was observed in peripheral lymphocytes but not healthy controls. A prior study revealed high PD-1/PD-L1expression in subjects with tumors than in healthy controls [73]. PD-L1 expression was associated with CD36 expression [74], and its enhancement was via CXCR3 in an Akt and STAT3-dependent manner [75]. The findings presuppose that CXCR3/CD36 and PDL1/PD-1 coexpression have a crucial role in lymphoma development, suggesting using this coexpression as a diagnostic test in managing lymphoma.

A significant increase in PD-1+CXCR3+% and PD-1+CXCR3+% was observed in subjects with extranodal involvement than in patients without extranodal involvement, giving evidence for the association between these cells and extranodal involvement's pathogenesis. CXCR3 expression in peripheral lymphocytes might fluctuate with the clinical outcome as T cell activation and differentiation regulates its expression [76]. The results might be the outcome of the immune system and lymphoma interaction, assuming that PD-L1/PD-1 coexpression with CXCR3 positive lymphocytes is engaged in tumor invasion and extranodal involvement in lymphoma. Another scenario might be due to the PD-L1+CXCR3 and PD-1+CXCR3 cell-mediated tolerance state, enabling extranodal involvement in lymphoma. PD-L1 and CXCR3 expressions were associated with tumor progression and a worse prognosis [67, 77].

High percentages of circulating PD+L1+CD36+ and PD-1+CD36+ lymphocytes were found in lymphoma subjects with extranodal involvement. CD36 contributed to tumor growth and progression [35] and was associated with PD-L1 expression [74]. Moreover, CD36 induced reprogramming of the lipid uptake in tumor cells, tumorigenesis, and metastasis [78]. CD36 blocking might be a potential new lymphoma therapy [79]. CD36 can attach to transmembrane proteins on the surface of cells, such as PD-L1/PD-1, which might induce signal transduction and ligand binding. Lymphoma progression and extranodal involvement in lymphomas could be initiated by PD-L1/PD-1 coexpression with CD36.

The findings revealed that lymphoma patients' peripheral CXCR3-positive lymphocytes have high PD-L1/PD-1 expression levels. CXCR3+PD-L1+%, on the other hand, was 5-6 times higher than CXCR3+PD-1+%. The data reveal that PD-L1, but not PD-1, is involved in lymphoma emergence by mediating extranodal involvement, assuming that PD-L1 might be the most significant molecule in the extranodal involvement of lymphoma. Previous studies demonstrated that CXCL10 could activate the p44/42 ERK and Akt signaling pathways and activate p38 MAPK in T lymphocytes, enhancing apoptosis [80]. According to the findings, the negative PD-L1/PD-1 signaling in circulating lymphocytes might be via CXCR3 or CD36. Another scenario is that CXCR3 and CD36 might increase PD-L1/PD-1 expression, enhancing CXCR3/CD36 and PD-L1/PD-1 crosstalk. The coexpression of these molecules may act as inhibitory molecules by inducing lymphocyte apoptosis, which may be linked to the pathogenesis and etiology of lymphoma extranodal involvement. PD-L1/PD-1 coexpression with CXCR3/CD36 could significantly impact clinical lymphoma activity.

In lymphoma patients without extranodal involvement, posttherapy PD-L1/PD-1 and CXCR3/CD36 coexpression were reduced, suggesting that chemotherapy might induce a disruption in PD-1/PD-L1 signaling. In patients who had achieved remission, PD-L1 expression was reduced [81]. By reducing PD-1 expression in peripheral lymphocytes and enhancing the immune response, chemotherapy may serve as an effective antitumor treatment [82]. Chemotherapy may increase the nuclear expression of PD-1/PD-L1 while decreasing the surface expression [83]. Low PD-L1/PD-1+CD36 percentages promote peripheral cell proliferation, providing a possible antitumor mechanism in lymphoma. The findings assumed that the relevance of immune cells in antitumor immunity could be determined by the decline in PD-L1/PD-1 coexpression with CXCR/CD36 in peripheral lymphocytes.

Compared to healthy controls, sera from lymphoma patients had a higher concentration of IL-19. The findings appeared to be consistent with prior studies that showed an elevation of IL-19 in patients with NHL compared to normal controls [42]. IL-19 levels were associated with high LDH levels. High LDH levels were associated with inferior prognosis in lymphoma [84]. In the current study, patients with extranodal involvement had significantly higher IL-19 levels than patients without extranodal involvement. Previous studies revealed IL-19 association with tumor metastasis and poor clinical outcomes [43, 44]. This refers to the significance of employing IL-19 to identify extranodal involvement, implying earlier treatment and increased endurance.

CXCR3+ and PD-L1/PD-1+CXCR3+ lymphocytes were significantly associated with high LDH levels. Furthermore, these cells were negatively associated with albumin levels, which agreed with previous reports [85]. This observation suggested a key role for CXCR3+, PD-L1+CXCR3+, and PD-1+CXCR3+ lymphocytes in lymphoma pathogenesis and progression.

The results indicated that CXCR3+, PD-L1+CXCR3+%, and PD-1+CXCR3+ lymphocytes were associated with poor prognostic features such as hepatomegaly, staging, splenomegaly, and recurrence. Previous studies reported the high expression of CXCR3, and PD-L1/PD-1 was related to cancer invasion [86–90]. PD-L1+CXCR3+ and PD-1+CXCR3+ lymphocytes might be involved in inferior prognosis in lymphoma.

In this current study, some clinical criteria were significantly associated with high IL-19 levels. Pretherapy IL-19 levels were significantly associated with both hepatomegaly and splenomegaly. Moreover, IL-19 levels had a significant correlation with lymphedema and recurrence. Previous studies showed the association between high IL-19 levels and inferior clinical outcomes [44], assuming a major role for IL-19 in lymphoma progression.

Bone marrow, lung, and lymph vessel involvement were positively correlated with CXCR3+%, PD-L1+CXCR3+%, and PD-1+CXCR3+% of cells. PD-L1+CXCR3+ cells majorly mediate the metastasis of melanoma and colon carcinoma [23, 24]. Interestingly, PD-L1+CD36+ and PD-1+CD36+ lymphocytes mediate lung and bone marrow involvement. Lymphoma prognosis is closely associated with bone marrow and lymph vessel involvement [3, 91]. To our knowledge, these findings have not been reported before. PD-L1/PD-1+CXCR3+ cells might represent a novel and critical prognostic marker as their expression was related to extranodal involvement and prognosis. A possible scenario is that PD-L1 and CXCR3 function as immune-suppressive agents in cancer [92, 93].

This study revealed that high IL-19 levels were associated with bone marrow and lymph vessel involvement. In former studies, IL-19 levels were related to poor prognosis and metastasis [94]. Interestingly, an association between lung involvement and higher serum IL-19 levels was observed in lymphoma. According to the findings, IL-19 could be a biomarker for extranodal lymphoma involvement.

This study linked high percentages of PD-L1+CXCR3+, PD-1+CXCR3+, and high IL-19 levels to shorter EFS. The results also assume that posttherapy PDL1+CD36+% and PD-1+CD36+ percentages are correlated with poor prognosis, considering prognostic biomarkers in lymphoma patients. High expression of PD-1/PD-1 is confined to poor prognosis in lymphoma [95]. PD-L1/PD-1 coexpression with CXCR3/CD36 and IL-19 might play an inferior prognostic role in lymphoma, providing a new significant era in lymphoma immunotherapy, especially in patients with extranodal involvement.

PD-L1/PD-1 coexpression with CXCR3/CD36 in identifying patients with extranodal involvement was assessed using ROC curves. The AUCs of the pre- and posttherapy PD-L1+CXCR3+% were 0.981 and 0.961, respectively, with high specificity and sensitivity. The cut-off values were >33 and >40. CXCR3+% and PD-L1+CXCR3+% yielded the best sensitivity and specificity. Moreover, the ROC curve was assessed for posttherapy PD-L1/PD-1+CD36+ percentages. The AUCs were 0.694 and 0.769, with reduced sensitivity and specificity. Thus, according to the findings, PD-L1/PD-1+CD36+% is insufficient for identifying extranodal involvement in lymphoma patients. Furthermore, the pretherapy IL-19 ROC curves demonstrated a pattern of extranodal involvement (*AUC* = 0.993 and *P* < 0.001). The specificity and sensitivity were 97.73% and 100% at a cut-off >280 pg/mL.

This study had some limitations: (1) the small size of the subjects in this study; (2) short follow-up time, longer follow-up, and multicenter collaborations are needed to confirm PD-L1/PD-1's role; (3) PD-L1/PD-1 coexpression with CXCR3/CD36 should be investigated in tumor tissue; (4) cell function activities such as differentiation, proliferation, apoptosis, and cytokine release were not carried out; future research will investigate these issues; (5) PD-L1/PD-1 coexpression with CXCR3/CD36 was not investigated in different lymphocyte subsets; further studies are required.

In conclusion, PD-L1/PD-1 coexpression with CXCR3/CD36 and serum IL-19 may be involved in lymphoma extranodal involvement and have prognostic and predictive values in lymphoma. The findings could also shed light on the role of circulating CXCR3 and CD36-positive lymphocyte cells in lymphoma. Future clinical trials and research are required to create new treatments based on PD-L1/PD-1-induced lymphoma immune evasion mechanisms and host immune response regulation. The combination of PD-L1/PD-1 blockades, anti-CXCR3/CD36, and IL-19 monoclonal antibody therapy might start a new era for immunotherapy. PD-L1+CXCR3+ lymphocytes and serum IL-19 might play a more important role in poor clinical behavior in lymphoma.

## Figures and Tables

**Table 1:** Extranodal involvement sites in 34 lymphoma patients.

		Patients with lymphoma
		*n* = 78
**Extranodal involvement**	No	44 (56.4%)
	Yes	34 (43.6%)
Central nervous system	No	77 (98.7%)
	Yes	1 (1.3%)
Bone marrow	No	52 (66.7%)
	Yes	26 (33.3%)
Lymph vessels	No	61 (78.2%)
	Yes	17 (21.8%)
Intestine	No	77 (98.7%)
	Yes	1 (1.3%)
Lung	No	68 (87.2%)
	Yes	10 (12.8%)
Liver	No	71 (91%)
	Yes	7 (9%)
Thyroid	No	77 (98.7%)
	Yes	1 (1.3%)
Spleen	No	70 (89.7%)
	Yes	8 (10.3%)

**Table 2:** IL-19 and PD-L1/PD-1 coexpression with CXCR3/CD36 in newly diagnosed lymphoma patients and healthy controls. IQR: interquartile range; pre-T: pretherapy. ^∗∗^ identifies highly significant differences *P* < 0.001.

		Controls	Lymphoma patients	*P* value
		*n* = 50	*n* = 78	
Pre-T IL-19 (pg/ml)	Median IQR	7.2 (4.4-10.9)	237 (86-510.5)	<0.001^∗∗^
Pre-T CXCR3+%	Median IQR	11.2 (9.7-13.2)	40 (35-65)	<0.001^∗∗^
Pre-T PDL-1+CXCR3+%	Median IQR	1.1 (1-1.5)	32 (25-54)	<0.001^∗∗^
Pre-T PD-1+CXCR3+%	Median IQR	0.7 (0.5-1)	10 (8-13)	<0.001^∗∗^
Pre-T CD36+%	Median IQR	4.2 (3.5-5.7)	13 (12-15)	<0.001^∗∗^
Pre-T CD36+PDL1+%	Median IQR	1 (0.5-1.4)	8 (6-9)	<0.001^∗∗^
Pre-T PD-1+CD36+%	Median IQR	0.2 (0.1-0.4)	5 (4-6)	<0.001^∗∗^

**Table 3:** PD-L1/PD-1 coexpression with CXCR3/CD36 and IL-19 in different lymphoma patients' outcomes. CR: complete remission; PR: partial remission; refractory: therapy resistance; post-T: posttherapy; IQR: interquartile range; pre-T: pretherapy. ^∗∗^ identifies highly significant differences *P* < 0.001; ^∗^ identifies significant differences *P* < 0.05.

		CR I	PR II	Relapse III	Refractory IV	I vs. II	I vs. III	I vs. IV	II vs. III	II vs. IV	III vs. IV
Pre-T IL-19 (pg/ml)	Median IQR	84 (65-104)	259 (178-389)	512 (325-703)	355 (162.5-627.5)	<0.001^∗∗^	<0.001^∗∗^	0.003^∗^	0.011^∗^	0.694	0.243
Pre-T CXCR3+%	Median IQR	35 (33-38)	40 (39-42)	66 (60-72)	40 (38.5-72)	<0.001^∗∗^	<0.001^∗∗^	0.005^∗^	<0.001^∗∗^	0.596	0.253
Pre-T PDL-1+CXCR3+%	Median IQR	25 (22-29)	32 (27-40)	55 (50-61)	29 (26.5-60.5)	0.008^∗^	<0.001^∗∗^	0.043^∗^	<0.001^∗∗^	0.600	0.242
Pre-T PD-1+CXCR3+%	Median IQR	9 (7-11)	11 (8-15)	12 (9-15)	10 (9-36)	0.017^∗^	0.001^∗^	0.092	0.553	0.629	0.876
Pre-T CD36+%	Median IQR	13 (12-14)	12 (10-15)	13 (11-15)	12 (10-13.5)	0.625	0.746	0.189	0.625	0.536	0.283
Pre-T CD36+PDL1+%	Median IQR	8 (6-9)	9 (8-11)	7 (6-9)	8 (8-8.5)	0.037^∗^	0.922	0.668	0.057	0.301	0.383
Pre-T PD-1+CD36+%	Median IQR	4 (3-5)	6 (5-7)	5 (3.8-5.3)	5 (4.5-6.5)	0.002^∗^	0.199	0.067	0.022^∗^	0.718	0.214
Post-T IL-19 (pg/ml)	Median IQR	40 (32-66)	219 (140-551)	560 (360-804)	480 (175.5-678.5)	<0.001^∗∗^	<0.001^∗∗^	0.005^∗^	0.018^∗^	0.359	0.421
Post-T CXCR3+%	Median IQR	24 (17-29)	45 (43-49)	78 (70-85)	44 (30-78.5)	<0.001^∗∗^	<0.001^∗∗^	0.026^∗^	0.013^∗^	0.793	0.153
Post-T PDL1+CXCR3+%	Median IQR	4 (2-5)	30 (27-40)	66 (50-74)	32 (14-63.9)	<0.001^∗∗^	<0.001^∗∗^	0.055	0.011^∗^	0.930	0.102
Post-T PD1+CXCR3+	Median IQR	1 (1-3)	13 (11-15)	11.5 (10.5-13)	12 (5.8-22.5)	<0.001^∗∗^	<0.001^∗∗^	0.029^∗^	0.115	0.895	0.715
Post-T CD36+%	Median IQR	59 (54-65)	45 (34-48)	27 (17-34)	41 (23-49.5)	<0.001^∗∗^	<0.001^∗∗^	0.010^∗^	0.002^∗^	0.631	0.091
Post-T PDL1+CD36+%	Median IQR	7 (5-10)	24 (19-28)	17 (9.3-24.2)	25 (8.4-27.5)	<0.001^∗∗^	<0.001^∗∗^	0.026^∗^	0.109	0.827	0.483
Post-T PD-1+CD36+%	Median IQR	1 (1-2)	6 (5-8)	5 (4.5-6)	7 (3.4-8.5)	<0.001^∗∗^	<0.001^∗∗^	0.002^∗^	0.097	0.860	0.308

**Table 4:** Association between CXCR3%, PD-L1+CXCR3+%, PD-1+CXCR3+%, IL-19, and laboratory and clinicopathological criteria in lymphoma. LDH: lactate dehydrogenase; Hb: hemoglobin; pre-T: pretherapy. ^∗^ identifies significant differences at *P* < 0.05; ^∗∗^identifies highly significant differences at *P* < 0.001.

	Pre-T CXCR3+%		Pre-T PDL-1+CXCR3+%		Pre-T PD-1+CXCR3+%		Pre-T IL-19	
	*r*	*P* value	*r*	*P* value	*r*	*P* value	*r*	*P* value
Age	-0.028	0.805	-0.023	0.843	0.003	0.978	0.025	0.827
Hb g/dL	-0.214	0.060	-0.254	0.025^∗^	-0.110	0.339	-0.057	0.621
L D H U/L	0.344	0.002^∗^	0.375	0.001^∗^	0.315	0.005^∗^	0.349	0.002^∗^
Albumin g/dL	-0.269	0.017^∗^	-0.326	0.004^∗^	-0.337	0.003^∗^	-0.124	0.278
Ascites	0.017	0.884	0.057	0.621	0.018	0.875	0.116	0.311
Lymphedema	0.452	<0.001^∗∗^	0.445	<0.001^∗∗^	0.283	0.012^∗^	0.426	<0.001^∗∗^
Hypertension	-0.120	0.294	-0.083	0.472	0.077	0.501	0.085	0.462
Diabetes	0.093	0.418	0.172	0.132	0.215	0.059	0.149	0.192
BCR-ABL	0.262	0.020^∗^	0.271	0.016^∗^	-0.044	0.702	0.135	0.240
Hepatomegaly	0.464	<0.001^∗∗^	0.398	<0.001^∗∗^	0.335	0.003^∗^	0.362	0.001^∗^
Splenomegaly	0.348	0.002^∗^	0.268	0.018^∗^	0.172	0.132	0.231	0.042^∗^
Stage	0.498	<0.001^∗∗^	0.437	<0.001^∗∗^	0.344	0.002^∗^	0.406	1
General lymphadenopathy	0.362	0.001^∗^	0.304	0.007^∗^	0.170	0.137	0.239	0.035^∗^
Recurrence	0.645	<0.001^∗∗^	0.676	<0.001^∗∗^	0.310	0.006^∗^	0.641	<0.001^∗∗^

**Table 5:** Correlations between PD-L1/PD-1 coexpression with CXCR3/CD36, IL-19, and different sites of extranodal involvement in lymphoma. Post-T: posttherapy; CNS: the central nervous system. ^∗^ identifies significant differences at *P* < 0.05; ^∗∗^ identifies high significant differences at *P* < 0.001.

	Post-T IL-19		Post-T CXCR3%		Post-T PDL-1 + CXCR3 + %		Post-T PD-1 + CXCR3 + %		Post-T CD36%		Post-T PD-L1 + CD36 + %		Post-T PD-1 + CD36 + %	
	*r*	*P* value	*r*	*P* value	*r*	*P* value	*r*	*P* value	*r*	*P* value	*r*	*P* value	*r*	*P* value
Metastasis	0.848	<0.001^∗∗^	0.771	<0.001^∗∗^	0.793	<0.001^∗∗^	0.528	<0.001^∗∗^	-0.792	<0.001^∗∗^	0.333	0.003^∗^	0.469	<0.001^∗∗^
CNS	0.023	0.843	0.149	0.192	0.175	0.126	0.048	0.674	-0.119	0.299	0.081	0.480	0.093	0.420
Bone marrow	0.637	<0.001^∗∗^	0.645	<0.001^∗∗^	0.676	<0.001^∗∗^	0.369	0.001^∗^	-0.633	<0.001^∗∗^	0.312	0.005^∗^	0.390	<0.001^∗∗^
Lymph vessels	0.437	<0.001^∗∗^	0.499	<0.001^∗∗^	0.487	<0.001^∗∗^	0.272	0.016^∗^	-0.386	<0.001^∗∗^	0.144	0.210	0.144	0.210
Intestine	-0.139	0.224	-0.142	0.216	-0.190	0.095	-0.150	0.189	0.167	0.144	-0.180	0.115	-0.195	0.086
Lung	0.278	0.014^∗^	0.277	0.014^∗^	0.286	0.011^∗^	0.235	0.038^∗^	-0.205	0.071	0.141	0.219	0.234	0.040^∗^
Liver	0.143	0.210	0.118	0.305	0.080	0.487	0.176	0.124	-0.071	0.538	0.129	0.262	0.111	0.332
Thyroid	0.165	0.150	0.160	0.163	0.188	0.100	0.048	0.674	-0.114	0.321	0.104	0.366	0.136	0.234
Spleen	0.124	0.280	0.103	0.368	0.148	0.197	0.043	0.711	-0.327	0.004^∗^	0.023	0.838	0.111	0.335

## Data Availability

The datasets created and analyzed during this study are not available to the public. They are, however, available from the corresponding author upon reasonable request.

## Abbreviations

CD36: Cluster of differentiation 36
CHOP: Cyclophosphamide, hydroxydaunorubicin, oncovin, and prednisone
CR: Complete remission
CT: Computed tomography
DLBCL: Diffuse large B cell lymphoma
ELISA: Enzyme-linked immune sorbent assay
FCL: Follicular cell lymphoma
GPCR: G protein-coupled cell surface receptor
IL-19: Interleukin-19
LDH: Lactate dehydrogenase
NHL: Non-Hodgkin lymphomas
PBS: Phosphate-buffered solution
PD-1: Programmed death-1
PD-L1: Programmed death-ligand 1
PET: Positron emission tomography
PR: Partial response
ROC: Receiver operating characteristic curve.

## Supplementary Materials

Supplementary Materials Table S1: characteristics of 78 lymphoma patients and 50 healthy volunteers. Table S2: characteristics of 44 lymphoma patients without extranodal involvement and 34 patients with extranodal involvement. Table S3: pretherapy and posttherapy PD-L1/PD-1 coexpression with CXCR3/CD36 in 34 lymphoma patients with extranodal involvement. Table S4: pretherapy and posttherapy PD-L1/PD-1 coexpression with CXCR3/CD36 in 44 patients without extranodal involvement. Figure S1: (a) lymphocytes gating is used to assess positive cells in a lymphoma patient with extranodal involvement; (b) Green P2 represents CXCR3+ lymphocytes; (c) upper right quadrant representing positive PD-L1+CXCR3+ cells. Figure S2: (a) lymphocytes gating is used to assess positive cells in a lymphoma patient with extranodal involvement; (b) Green P2 represents CXCR3+ lymphocytes; (c) upper right quadrant representing positive PD-1+CXCR3+ lymphocytes. Figure S3: (a) lymphocytes gating is used to assess positive cells in a lymphoma patient with extranodal involvement; (b) Green P2 represents CD36+ lymphocytes; (c) upper right quadrant representing positive PD-L1+CD36+ lymphocytes. Figure S4: (a) lymphocytes gating is used to assess positive cells in a lymphoma patient with extranodal involvement; (b) Green P2 represents CD36+ lymphocytes; (c) upper right quadrant representing positive PD-1+CD36+ lymphocytes.
