# Supplementary material for: Coexpression of PD-L1/PD-1 with CXCR3/CD36 and IL-19 Increase in Extranodal Lymphoma
Source: J Immunol Res. 2023 Jan 23;2023:4556586. doi: 10.1155/2023/4556586 (PMC9886470; doi:10.1155/2023/4556586)
Supplement: Supplementary Materials — Table S1: characteristics of 78 lymphoma patients and 50 healthy volunteers. Table S2: characteristics of 44 lymphoma patients without extranodal involvement and 34 patients with extranodal involvement. Table S3: pretherapy and posttherapy PD-L1/PD-1 coexpression with CXCR3/CD36 in 34 lymphoma patients with extranodal involvement. Table S4: pretherapy and posttherapy PD-L1/PD-1 coexpression with CXCR3/CD36 in 44 patients without extranodal involvement. Figure S1: (a) lymphocytes gating is used to assess positive cells in a lymphoma patient with extranodal involvement; (b) Green P2 represents CXCR3+ lymphocytes; (c) upper right quadrant representing positive PD-L1+CXCR3+ cells. Figure S2: (a) lymphocytes gating is used to assess positive cells in a lymphoma patient with extranodal involvement; (b) Green P2 represents CXCR3+ lymphocytes; (c) upper right quadrant representing positive PD-1+CXCR3+ lymphocytes. Figure S3: (a) lymphocytes gating is used to assess positive cells in a lymphoma patient with extranodal involvement; (b) Green P2 represents CD36+ lymphocytes; (c) upper right quadrant representing positive PD-L1+CD36+ lymphocytes. Figure S4: (a) lymphocytes gating is used to assess positive cells in a lymphoma patient with extranodal involvement; (b) Green P2 represents CD36+ lymphocytes; (c) upper right quadrant representing positive PD-1+CD36+ lymphocytes. [file 4556586.f1.docx]

|  |  | **Control** | **Cases** | **P value** |
| --- | --- | --- | --- | --- |
|  |  | **N=50** | **N=78** |  |
| Age (years) | *Range*  *Mean ± SD* | (24-81)  44.7±15.2 | (11-81)  42.3±16.7 | 0.409 |
| Sex | Male  Female | 36(72%)  14(28%) | 39(50%)  39(50%) | 0.014* |
| HCV.cat | No  Yes | 50(100%)  0(0%) | 53(67.9%)  25(32.1%) | <0.001* |
| Ascites | No  Yes | 50(100%)  0(0%) | 74(94.9%)  4(5.1%) | 0.104 |
| Lymphedema | No  Yes | 50(100%)  0(0%) | 60(76.9%)  18(23.1%) | <0.001* |
| Hypertension | No  Yes | 50(100%)  0(0%) | 70(89.7%)  8(10.3%) | 0.019* |
| Diabetes | No  Yes | 50(100%)  0(0%) | 59(75.6%)  19(24.4%) | <0.001* |
| BCR ABL.cat | No  Yes | 50(100%)  0(0%) | 71(91%)  7(9%) | 0.029* |
| Hepatomegaly | No  Yes | 50(100%)  0(0%) | 44(56.4%)  34(43.6%) | <0.001* |
| Splenomegaly | No  Yes | 50(100%)  0(0%) | 37(47.4%)  41(52.6%) | <0.001* |

**Table S1:** Characteristics of 78 patients with lymphoma and 50 healthy volunteers. HCV: Hepatitis C virus. * Identifies significant Differences *p* <0.05; ** identifies highly significant differences *p* < 0.001.

|  |  | **Without extra-nodal involvement** | **With**  **extra-nodal involvement** | | P-value |
| --- | --- | --- | --- | --- | --- |
|  |  | **N=44** | **N=34** | |  |
| Diagnosis | Non-Hodgkin’s Lymphoma  Hodgkin’s Lymphoma | 41(93.2%)  3(6.8%) | 30(88.2%)  4(11.8%) | | 0.693 |
| Subtype | Diffuse large B cell lymphoma  Chronic lymphocytic leukemia  Follicular cell lymphoma  Mucosa-associated lymphoid tissues  Marginal zone lymphoma  Small Lymphocytic Lymphoma  Nodular sclerosing | 28(63.6%)  3(6.8%)  7(15.9%)  2(4.5%)  1(2.3%)  0(0%)  3(6.8%) | 24(70.6%)  4(11.8%)  1(2.9%)  0(0%)  0(0%)  1(2.9%)  4(11.8%) | | 0.216 |
| Stage^¶^ | Stage I  Stage II  Stage III  Stage IV | 6(13.6%)  23(52.3%)  8(18.2%)  7(15.9%) | 1(2.9%)  4(11.8%)  9(26.5%)  20(58.8%) | | <0.001* |
| Recurrence | No  Yes | 41(93.2%)  3(6.8%) | | 9(26.5%)  25(73.5%) | <0.001* |
| Death | No  Yes | 41(93.2%)  3(6.8%) | | 27(79.4%)  7(20.6%) | 0.018* |
| Age (years) | *Range*  *Mean ± SD* | (18-75)  42±16.1 | | (11-81)  42.8±17.6 | 0.827 |
| Sex | Male  Female | 17(38.6%)  27(61.4%) | | 22(64.7%)  12(35.3%) | 0.039* |

**Table S2:** Characteristics of 44 lymphoma patients without extra-nodal involvement and 34 lymphoma patients with extra-nodal involvement. *Identifies significant Differences *p* <0.05; ** identifies highly significant differences *P* < 0.001. ^¶^ Ann Arbore’s stages for lymphoma types and Rai staging for CLL.

|  |  | **Pre-therapy** | **Post-therapy** | **P-value** |
| --- | --- | --- | --- | --- |
|  |  | **N=34** | **N=34** |  |
| CXCR3+% | *Median*  *IQR* | 66  (59.5-71.3) | 78  (72.8-85) | 0.002* |
| PDL-1+CXCR3+% | *Median*  *IQR* | 55  (50-60.3) | 65.5  (58.8-71.3) | 0.003* |
| PD-1+CXCR3+% | *Median*  *IQR* | 12  (9.8-15.3) | 12.2  (11-14) | 0.0623 |
| CD36+% | *Median*  *IQR* | 13  (11.8-15) | 27  (16.3-33.1) | <0.001** |
| CD36+PDL1+% | *Median*  *IQR* | 8  (6.8-9) | 22  (9.5-25.3) | <0.001** |
| PD-1+CD36+% | *Median*  *IQR* | 5  (4-6) | 5.8  (4.9-6) | 0.029* |

**Table S3:** **Pre-therapy and post-therapy PD-L1/PD-1 co-expression with CXCR3/CD36 in 34 lymphoma patients with extra-nodal involvement**. IQR: interquartile range. *Identifies significant Differences *p* <0.05; ** identifies highly significant differences p < 0.001.

|  |  | **Pre-therapy** | **Post-therapy** | **P-value** |
| --- | --- | --- | --- | --- |
|  |  | **N=44** | **N=44** |  |
| CXCR3+% | *Median*  *IQR* | 36  (33-39) | 26  (18-42.5) | <0.001** |
| PDL-1+CXCR3+% | *Median*  *IQR* | 25  (22-29) | 4  (2.3-27) | 0.072 |
| PD-1+CXCR3+% | *Median*  *IQR* | 9  (7-11) | 2  (1-11.8) | <0.001** |
| CD36+% | *Median*  *IQR* | 12  (12-15) | 55.5  (48.3-63) | <0.001** |
| CD36+PDL1+% | *Median*  *IQR* | 8  (6-9) | 8  (6-21.3) | <0.001** |
| PD-1+CD36+% | *Median*  *IQR* | 4  (3-6) | 1  (1-5) | 0.002* |

**Table S4: Pre-therapy and post-therapy PD-L1/PD-1 co-expression with CXCR3/CD36 in 44 patients without extra-nodal involvement.** IQR: inter quartile range. *Identifies significant Differences *p* <0.05; ** identifies highly significant differences *p* < 0.001.


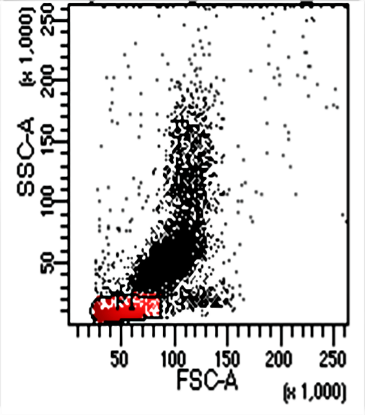

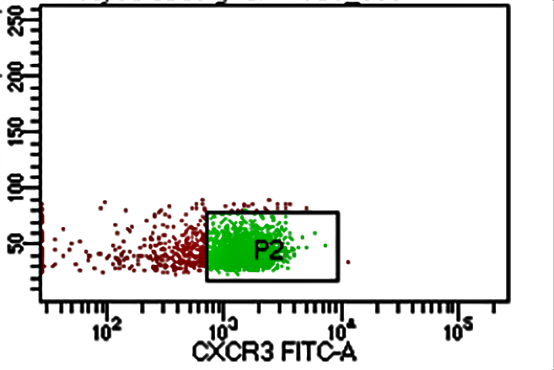

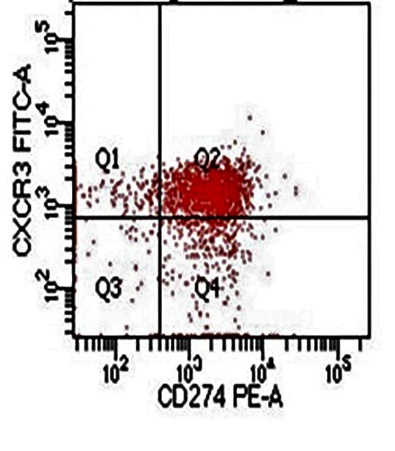


**a**

**b**

**c**

**78%**

**Figure S1:** (a) Lymphocytes Gating is used to assess positive cells in a lymphoma patient with extra-nodal involvement. (b) Green P2 represents CXCR3+ lymphocytes. (c) upper right quadrant representing positive PD-L1+CXCR3+ lymphocytes.


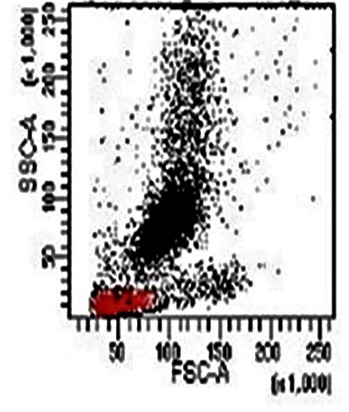

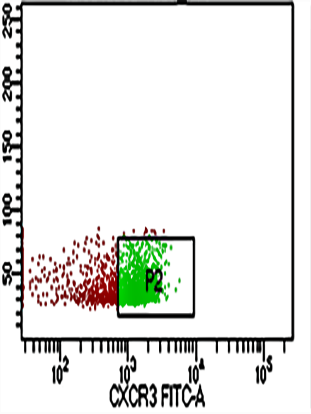

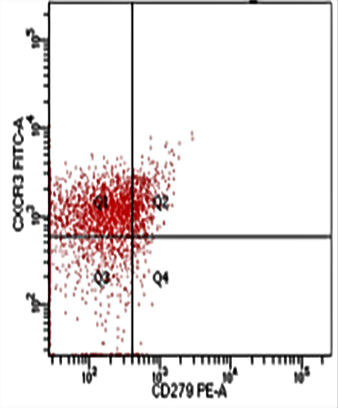


**a**

**b**

**c**

**14%**

**Figure S2:** (a) Lymphocytes Gating is used to assess positive cells in a lymphoma patient with extra-nodal involvement. (b) Green P2 represents CXCR3+ lymphocytes. (c) upper right quadrant representing positive PD-1+CXCR3+ lymphocytes. (d) Green cells representing PD-1+CXCR3+ lymphocytes.


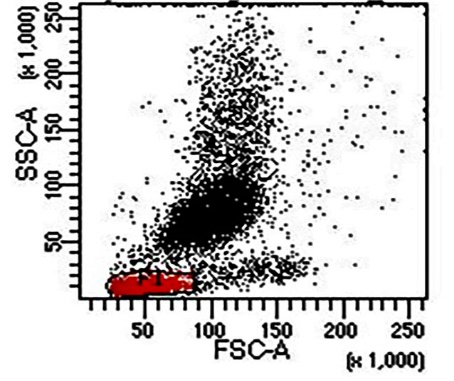

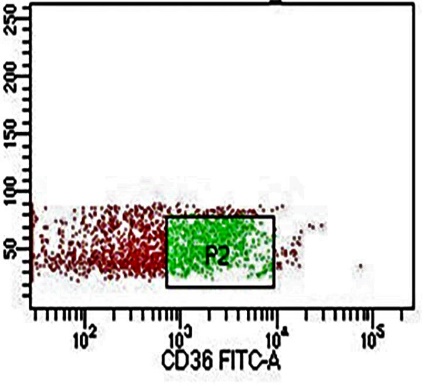

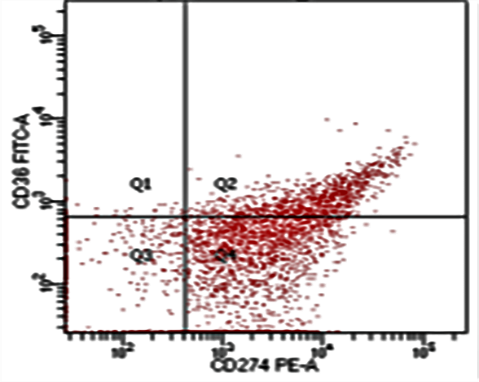


**a**

**b**

**c**

**Figure S3:** (a) Lymphocytes Gating is used to assess positive cells in a lymphoma patient with extra-nodal involvement. (b) Green P2 represents CD36+ lymphocytes. (c) upper right quadrant representing positive PD-L1+CD36+ lymphocytes.


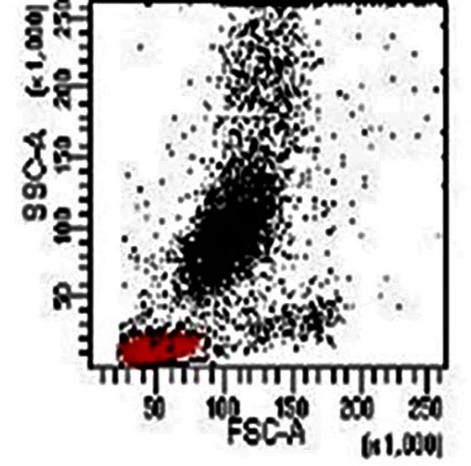

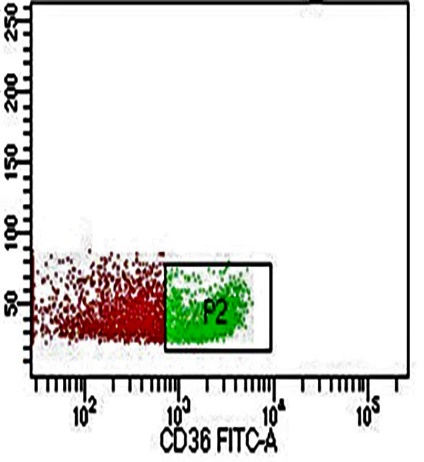

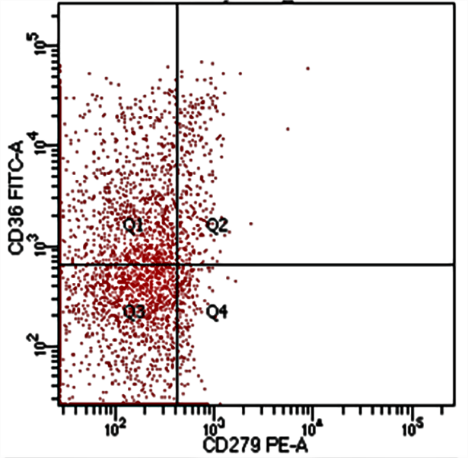


**c**

**b**

**a**

**6%**

**Figure S4:** (a) Lymphocytes Gating is used to assess positive cells in a lymphoma patient with extra-nodal involvement**.** (b) Green P2 representing CD36+ lymphocytes. (c) upper right quadrant representing positive PD-1+CD36+ lymphocytes.
